# Hydrothermal Treatment of Wheat Bran under Mild Acidic or Alkaline Conditions for Enhanced Polyphenol Recovery and Antioxidant Activity

**DOI:** 10.3390/molecules29061193

**Published:** 2024-03-07

**Authors:** Eirini Papadaki, Spyros Grigorakis, Dimitrios Palaiogiannis, Stavros I. Lalas, Paraskevi Mitlianga

**Affiliations:** 1 Laboratory of Food Chemistry and Technology, Department of Chemical Engineering, University of Western Macedonia, ZEP Campus, 50100 Kozani, Greece; pmitliagka@uowm.gr; 2 Department of Food Quality & Chemistry of Natural Products, Mediterranean Agronomic Institute of Chania (M. A. I. Ch.), International Centre for Advanced Mediterranean Agronomic Studies (CIHEAM), P.O. Box 85, 73100 Chania, Greece; 3 Department of Food Science & Nutrition, University of Thessaly, N. Temponera Street, 43100 Kardits, Greece

**Keywords:** wheat bran, polyphenols, acid catalysis, alkaline catalysis, antioxidants, ferulic acid, hydrothermal treatment, process severity

## Abstract

This study was undertaken to investigate the effects of hydrothermal treatments under mild acid and alkaline conditions on polyphenol release and recovery from wheat bran (WB). After an initial screening of various food-grade substances, strong evidence was raised regarding the potency of citric acid and sodium carbonate to provide WB extracts exceptionally enriched in polyphenols. Thus, these two catalysts were tested under various time and temperature combinations, and the processes were described by linear models based on severity factor. The most effective treatments were those performed with 10% of either citric acid or sodium carbonate, at a constant temperature of 90 °C for 24 h, providing yields in total polyphenols of 23.76 and 23.60 mg g^−1^ dry mass of ferulic acid equivalents, respectively. Liquid chromatography–mass spectrometry analyses revealed that, while the sodium carbonate treatment afforded extracts enriched in ferulic acid, treatments with citric acid gave extracts enriched in a ferulate pentose ester. The extracts produced from those treatments also exhibited diversified antioxidant characteristics, a fact ascribed to the different polyphenolic composition. To the best of the authors’ knowledge, this is the first report demonstrating the effective release of ferulic acid and a ferulate pentose ester from WB, using benign acid and alkali catalysts, such as citric acid and sodium carbonate.

## 1. Introduction

There is currently an ever-increasing awareness concerning the irrational overexploitation of natural resources that leads to their depletion. At the same time, waste generation from various industrial activities has become a prime issue with regard to environmental protection, public health, and land conservation. Particularly for the agri-food sector, there is an explicit diagnosis that it is a major contributor in generating highly polluting side-streams and processing residues, whose uncontrolled disposal and improper handling may have detrimental consequences on the environment. The acknowledgement of this fact has shifted strategies toward adopting sustainable production models, within various circular economy frames [1,2], which aim at valorizing food processing wastes as raw materials for the production of bio-fuels, energy, value-added chemicals, and platform molecules [3].

The recovery of bioactive substances from food waste biomass is a prospect of high priority, as manifested by the outstanding ongoing research on this field. Several abundant plant food processing wastes may contain an array of phytochemicals, such as essential oils, polysaccharides, carotenoids, and polyphenols, which can be effectively recovered and used as cosmetic ingredients, food supplements, and pharmaceutical formulations [4]. Polyphenols constitute a distinct family of phytochemicals, which encompasses several classes, including phenolic acids, flavonoids, tannins, anthocyainin pigments, etc. [5]. Numerous substances belonging to these classes have been tested and identified as agents with antioxidant, anti-inflammatory, antimicrobial, cardioprotective, and anticancer properties [6]. On these grounds, there has been extensive research on natural sources where polyphenolic substances could be effectively retrieved from with minimal cost. Therefore, it is not surprising that certain plant food processing side-streams are under scrutiny in this regard, by virtue of their overwhelming polyphenolic load [7].

The cultivation and processing of cereals is of paramount significance for large populations around the globe, as they constitute precious and irreplaceable sources of both food and feed [8]. They comprise an important part of the food industry, while crops including maize, wheat, rice, and barley account for about 90% of cereal consumption. Cereal processing results in the generation of an overwhelming volume of processing residues which, owed to their constituents, may be considered as highly suitable raw materials for biorefinery purposes [8,9]. Wheat is one of the largest cereal crops around the globe, and it is primarily processed to flour, with a yield of approximately 73–77%. The remaining 23–27% consists mainly of wheat bran (WB), containing smaller proportions of endosperm and germ. WB accounts for nearly 25% of the grain weight, and it is by far the principal wheat processing by-product. There have been estimations that almost 150 million tons of WB are produced annually [10], and, thus, WB could be regarded as a biomaterial to produce value-added substances, such as antioxidant polyphenols [10,11].

Ferulic acid is largely the major polyphenolic compound occurring in WB, known for its antioxidant activity [12], but also its beneficial effects against other degenerative diseases, such as cancer and cardiovascular disorders [13]. By virtue of these properties, ferulic acid may be considered a biomolecule with strong prospects as a functional food ingredient, food antioxidant, and bio-vanillin precursor [14]. Ferulic acid occurs in WB as a constituent of both arabinoxylan and lignocellulosic complexes, with attachments being via ether or ester linkages [15], and its extraction from WB using conventional extraction techniques is particularly low. Thus, the effective recovery of ferulic acid under conditions routinely used in a solid–liquid extraction is not possible. Ferulic acid liberation and recovery may be boosted with acid or alkaline catalysis [16,17], but most methodologies deployed toward this direction are rather harsh, involving the use of environmentally aggravating chemicals, such as sulfuric acid and sodium hydroxide [18].

Hydrothermal treatment, that is, the use of aqueous media at relatively high temperatures, has recently been proven to contribute significantly to ferulic acid liberation from wheat bran [19]. On the other hand, other studies have shown that increased polyphenol recovery from WB could be achieved with hydrothermal treatment at notably lower temperature [20]. Furthermore, recent evidence emerged from a study on ultrasound-assisted polyphenol recovery from WB, showing that acid catalysis using citric acid may contribute to achieving increased total polyphenol yields, compared to water or water/ethanol extraction [21]. Based on these observations, this examination was undertaken to investigate the role of benign media in catalyzing the recovery of polyphenolic compounds from WB. With this purpose, a series of food-grade organic acids, but also organic and inorganic salts, were tested as acid and alkali catalysts. Performance screening was carried out by estimating the total polyphenol yield, while the composition of the extracts was profiled by employing liquid chromatography–mass spectrometry. Further extract assessment was accomplished by determining their antioxidant activity. To the best of the authors’ knowledge, such a study in this field is heretofore unreported, where sodium carbonate and citric acid are explicitly proposed as highly effective, non-corrosive catalysts for the production of extracts enriched in polyphenols (ferulic acid and ferulates).

## 2. Results and Discussion

### 2.1. Effect of Mild Acid Catalysis

The evidence that emerged from a previous investigation indicated that aqueous solutions of citric acid might enhance polyphenol extraction from WB upon heating at 90 °C for over 6 h [21]. This observation formed the basis to examine the role of citric acid concentration in polyphenol extraction, but also the efficacy of other common, natural organic acids, including acetic acid and lactic acid. Thus, WB was treated at 90 °C using aqueous solutions of acetic acid (AcA), lactic acid (LA), and citric acid (CA), with variable acid concentration. The concentrations employed, as well as the pH of each of the extraction media used, are analytically given in Table 1.

To obtain a more integrated image regarding the course of the extraction, sampling was accomplished at 3, 6, and 24 h (Table 2).

As can be seen, for all acids tested, increasing the concentration from 0.5 to 5.0% (*w*/*v*) was favorable in obtaining higher Y_TP_, and in the cases examined, extraction with 5% acid gave significantly increased Y_TP_ (*p* < 0.05). Likewise, it was shown that prolonging resident time up to 24 h resulted in higher Y_TP_. This finding suggested that the extended extraction period did not provoke any polyphenol loss, but rather, contributed to attaining increased recovery. It was also pointed out that significantly higher Y_TP_ values compared to water (*p* < 0.05) were achieved with any acid tested, but in most cases for an acid concentration of 5%, irrespective of the treatment duration. For the extraction with both acetic acid and lactic acid, the highest Y_TP_ values were 4.13 and 5.82 mg FAE g^−1^ DM, respectively, achieved with a concentration of 5%, at 24 h. The most efficacious extraction was performed with 5% citric acid, at 24 h, which afforded 18.77 mg FAE g^−1^ DM and significantly surpassed the efficiency of the processes performed with either acetic or lactic acid (*p* < 0.05). This outcome highlighted the exceptional potency of citric acid for polyphenol extraction from WB.

The effect of both the concentration and type of acid in the aqueous polyphenol extraction has been emphasized by earlier studies, yet discrepancies have been observed concerning the effects of different acids. For example, it has been suggested that flavanol extraction from red grape pomace may be more favorable using aqueous solutions of acetic acid compared to citric acid solution [22]. In that study, a significant role was also ascribed to acid concentration. A more thorough approach employing response surface methodology demonstrated that aqueous lactic acid media may be more effective for the extraction of flavonoids from red grape pomace, compared to citric, acetic, and tartaric acids [23].

However, aqueous citric acid solutions were highly suitable for anthocyanin extraction from red grape peels [24] and levodopa extraction from Mucuna pruriens [25]. On the other hand, other examinations reported that citric acid addition to aqueous media had no significant contribution in polyphenol recovery from blackberry residues, using a high-pressure hydrothermal technique [26]. In the same line, a more recent investigation revealed that the hydrothermal treatment of saffron processing wastes with pressurized aqueous solutions of citric and lactic acids offered no advantage over treatment with deionized water, with respect to polyphenol extraction [27].

### 2.2. Effect of Mild Alkaline Catalysis

Extractions with mild alkaline media were also performed, to juxtapose the results with those obtained with the acid-catalyzed treatments. For this reason, sodium acetate, trisodium citrate and sodium carbonate were chosen, to cover a pH range from 7.35 to 11.49 (Table 1). The use of alkaline media was deemed necessary, in the light of earlier data which suggested that WB hydrolysis under alkaline conditions afforded differentiated yields and polyphenolic profile compared to acid hydrolysis [17,28]. For the alkaline media, the pattern concerning the effect of both concentration and time on Y_TP_ was quite different than that seen with the acidic media. Particularly for trisodium citrate (TSC), the increase in concentration from 0.5 to 5% had a rather negative effect on Y_TP_, irrespective of the extraction duration (Table 3), and the use of 5% trisodium citrate resulted in obtaining significantly lower Y_TP_ (*p* < 0.05).

For the treatments with sodium acetate (SA) solutions, fluctuations were observed in Y_TP_ as a function of both concentration and residence time, and extraction with 1 and 5% sodium acetate for 24 h provided the same Y_TP_. For the treatments with 5% of either trisodium citrate or sodium acetate, no benefit was seen compared to treatments with water, since the yields attained were lower (*p* < 0.05). On the contrary, 5% sodium carbonate (SCar) solutions were proven to be highly effective, giving a Y_TP_ of 22.33 mg FAE g^−1^ DM, at 24 h. In fact, the yields obtained using sodium carbonate were always significantly higher than those achieved with either trisodium citrate of sodium acetate, irrespective of the sodium carbonate concentration or treatment duration (*p* < 0.05). In addition, the recovery achieved with 5% sodium carbonate at 24 h was almost 6.9 times higher than that obtained with neat water. These results pointed clearly to the high effectiveness of sodium carbonate solutions in the recovery of polyphenols from WB.

### 2.3. Comparative Extraction Efficiency Appraisal

**Figure 1:**
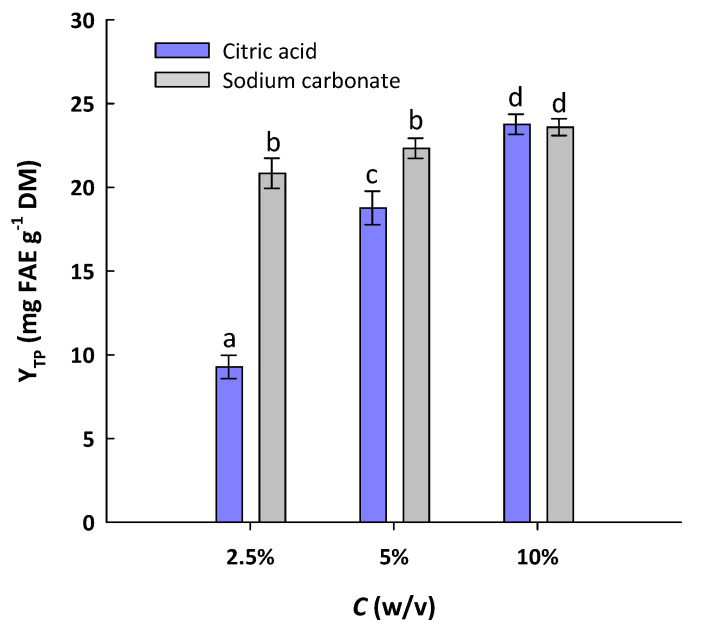
Yield in total polyphenols (Y_TP_) achieved after hydrothermal treatment of WB for 24 h at 90 °C, with citric acid and sodium carbonate solutions. Bars denote standard deviation (sd). Values designated with different letters (a, b, c, and d) are statistically different (*p* < 0.05).

As mentioned above, extraction media containing either citric acid or sodium carbonate exhibited an increasing efficiency as a function of concentration, and at 24 h, the most efficient were those with concentrations of 5%. To ascertain whether a further increase in concentration of either citric acid or sodium carbonate could afford even higher Y_TP_, solutions with 10% concentration were also tested. In Figure 1, it can be seen that at a concentration of 2.5%, sodium carbonate treatment was far more efficacious than the citric acid one. At 5%, sodium carbonate was still more effective, giving significantly higher yield (*p* < 0.05); yet, switching the concentration of both citric acid and sodium carbonate from 5 to 10% boosted polyphenol recovery to a significant extent (*p* < 0.05). These results indicated that (i) there was a benefit from the increase in citric acid and sodium carbonate concentration from 5 to 10%, and (ii) in both cases, the maximum Y_TP_ values were alike, suggesting that the treatment under the conditions employed resulted in a rather exhaustive polyphenol recovery. It should also be mentioned that compared to the treatment with deionized water, which at 24 h afforded a Y_TP_ of 3.25 mg FAE g^−1^ DM (Table 1), the treatment with 10% of either citric acid or sodium carbonate was almost 7.3 times more efficient. This finding illustrated the striking effect of citric acid and sodium carbonate on the hydrothermal treatment of WB to recover polyphenols.

The Y_TP_ values attained with 10% citric acid and sodium carbonate were 23.76 and 23.60 mg FAE g^−1^ DM, respectively. These yields may be considered exceptionally high, in the light of recent studies that reported levels of 19.76 ± 0.76 mg FAE g^−1^ DM, achieved with hydroethanolic solutions and sodium hydroxide catalysis [29]. In general, it has been demonstrated that the conventional extraction of WB with commonly used solvents could afford only low Y_TP_ levels, i.e., 0.84 mg FAE g^−1^ DM for aqueous ethanol extraction [30], 4.66 mg GAE g^−1^ DM for 50% acetone extraction [31], and 5.90 mg caffeic acid equivalents g^−1^ DM for deep eutectic solvent extraction [32]. Significantly higher Y_TP_ values, i.e., 12.20 mg FAE g^−1^ DM, have been reported in studies that employed more severe conditions, such as alkaline hydrolysis with sodium hydroxide [33]. However, treatment involving steam explosion was shown to be of outstanding efficiency, giving 27.71 mg GAE g^−1^ DM [34]. Furthermore, organosolv treatments with deep eutectic solvents, combined with ultrasonication pretreatment, were reported to afford yields as high as 94.62 mg FAE g^−1^ DM [21].

### 2.4. Severity Effects

Since the treatments carried out with 2.5, 5, and 10% of either citric acid or sodium carbonate were demonstrated to be of higher performance with respect to polyphenol recovery, these treatments were chosen to examine the effect of severity on total polyphenol yield, considering the time regimes of 3, 6, and 24 h (180, 300, and 1440 min, respectively). To this end, severity was estimated from CSF’, using Equation (5), to encompass the pH of the treatment, which is considered a significant contributor to the severity of a process [35]. The values of CSF’ determined in each case, along with the corresponding Y_TP_ values, are analytically presented in Table 4.

Considering CSF, which represents a rather fairer comparison of the treatment severities [35], the achievement of significantly higher Y_TP_ in the citric acid treatment would require at least a CSF’ of 7.67. Raising CSF’ from 7.67 to 8.37 did afford a significantly higher Y_TP_. In a recent study, where sulfuric acid was used as the acid catalyst, the maximum Y_TP_ found was 10.96 mg FAE g^−1^ DM, achieved at an CSF’ of 7.93 [29]. Based on this finding, it could be argued that the treatment carried out with 10% citric acid was far more effective, with lower severity. Likewise, the treatment performed with sodium carbonate gave significantly higher YTP at a CSF’ of 7.21, which was even lower than 8.37 required for efficient citric-acid-catalyzed treatment. This finding raised evidence that alkaline conditions might enable high polyphenol recovery yields, under less severe conditions. This hypothesis was in line with a previous examination, where it was demonstrated that the sodium-hydroxide-catalyzed hydrothermal treatment of WB had outstanding effectiveness in releasing polyphenols compared to the sulfuric-acid-catalyzed one [29].

**Figure 2:**
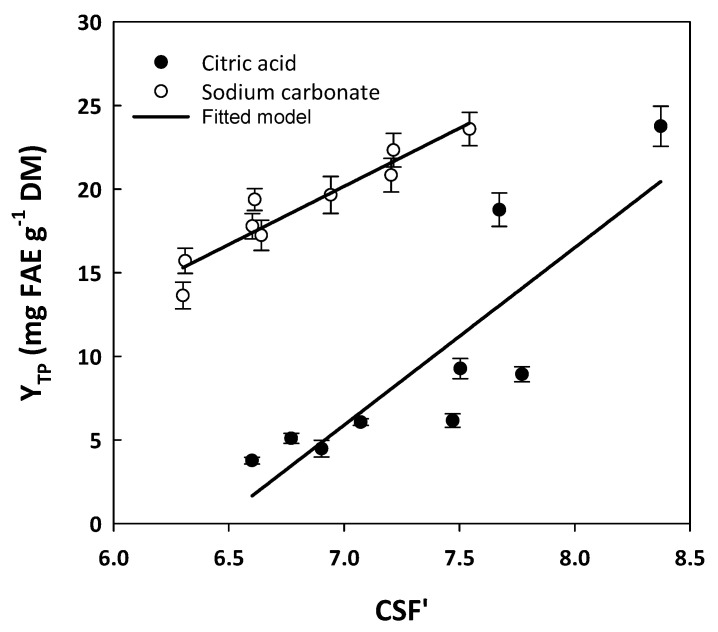
Linear regressions between the alternative severity factor (CSF’) values of the hydrothermal treatments performed, with the corresponding total polyphenol yield (Y_TP_) values.

To have a more thorough image of the effect of citric acid and sodium carbonate on polyphenol release, CSF’ values were plotted against Y_TP_ to identify any possible relationships (Figure 2). In the case of treatments with citric acid, the correlations established with linear regression gave statistically significant linear models, described by the following equations:

Y_TP(CA)_ = 10.60CSF’_CA_ − 68.34 (R^2^ = 0.73, *p* = 0.0032) (1)

Similarly, the models derived from the treatments with sodium carbonate were as follows:

Y_TP(SCar)_ = 6.95CSF’_SCar_ − 28.49 (R^2^ = 0.90, *p* < 0.0001) (2)

On the grounds of these correlations, it was made clear that there was a linear trend linking the severity of the treatments with the yield in total polyphenols. This trend was more pronounced in the case of Equation (2), which gave higher R^2^. Based on this outcome, it could be argued that increases in severity, within the limits tested in this investigation, were favorable in obtaining higher total polyphenol yields. This finding was in accordance with previous examinations on WB polyphenol extraction with hydroethanolic mixtures and pressurized liquid extraction, which demonstrated that severity was linked to total polyphenol yield with a linear and statistically significant correlation [36]. Additionally, more recent examinations on ethanol organosolv WB treatment showed that Y_TP_ displayed statistically significant linear correlation with CSF’ only when the process was acid-catalyzed, whereas for the alkali-catalyzed process, such a relationship was significant when the modified severity factor (MSF) was considered [29]. In that study, maximum Y_TP_ was achieved with alkaline catalysis, at CSF’ of 7.63.

### 2.5. Polyphenolic Composition—Tentative Polyphenol Release Mechanism

A critical appraisal of the results obtained from the hydrothermal treatments with either citric acid or sodium carbonate would dictate that both mild acidic and alkaline conditions boosted polyphenol recovery yield. Apparently, the effect of both treatments should be attributed to the ability of citric acid and sodium carbonate to catalyze liberation of bound phenolics from complex lignocellulosic matrices through acid- and alkaline-catalyzed hydrolysis [37]. To test this hypothesis, the extracts obtained with 10% citric acid, 10% sodium carbonate, and the control extracts obtained with water, 60% ethanol, and after the reference alkaline hydrolysis, were analyzed by liquid chromatography–mass spectrometry.

**Figure 3:**
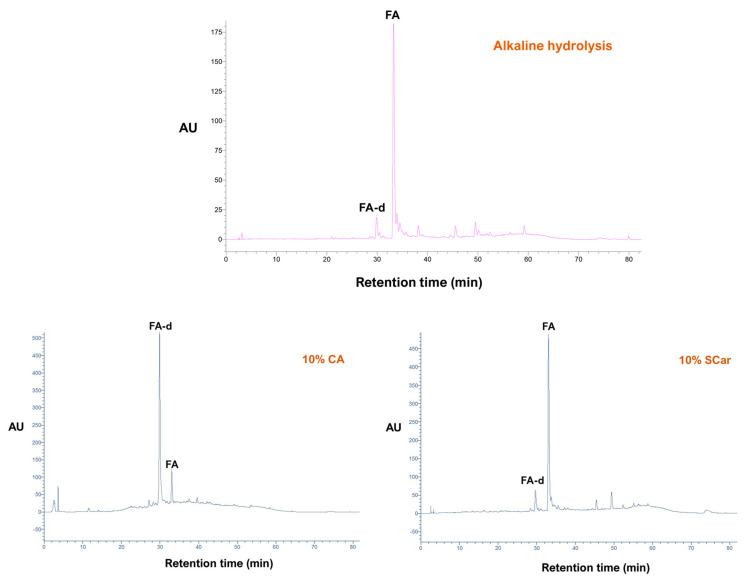
Chromatographic traces recorded at 320 nm of the WB extract produced with the reference method (alkaline hydrolysis), and of those generated with hydrothermal treatment using 10% citric acid (CA) and 10% sodium carbonate (SCar). Peak assignment: FA, ferulic acid; FA-d, ferulic acid derivative.

**Figure 4:**
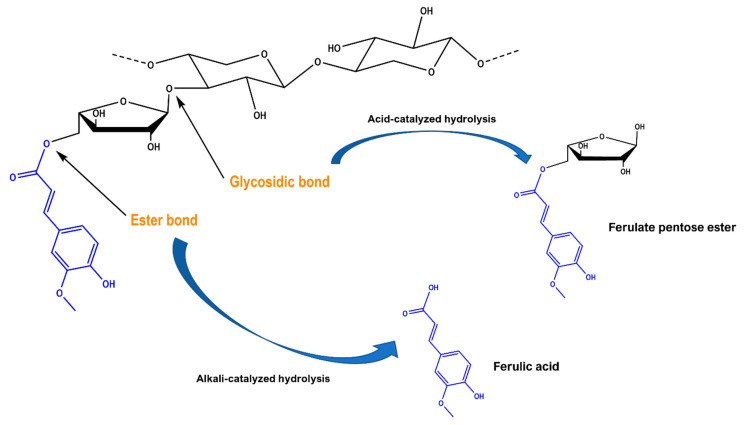
Putative mechanism of the generation of ferulic acid and ferulate pentose ester, following hydrothermal treatment with 10% sodium carbonate (alkali-catalyzed hydrolysis) and 10% citric acid (acid-catalyzed hydrolysis), respectively.

As can be seen in Figure 3, alkaline hydrolysis resulted in an extract enriched in ferulic acid (FA), which, based on earlier studies, was the anticipated outcome [29]. However, *p*-coumaric acid (*p*-CA), which may usually accompany FA, was not detected. The chromatogram of the extract produced with the sodium carbonate treatment gave an almost identical profile, which demonstrated that sodium carbonate had the same effect as sodium hydroxide. By contrast, the extract obtained from the citric acid treatment had a diversified composition, and the predominant peak was a substance eluted at 30 min. This peak displayed almost identical UV-vis spectrum with ferulic acid, providing evidence that it corresponded to a ferulate derivative (FA-d). The mass spectrum of FA-d obtained in the positive ion mode showed a molecular ion at *m*/*z* = 327, consistent with a ferulate pentose ester, as depicted in Figure 4. This was corroborated by the presence of the ion at *m/z* = 309, which corresponded to a dehydration product [M − 18]^+^, and the existence of the ion with *m/z* = 349, which represented an adduct with Na^+^ [M + 23]^+^. On these grounds, the structure of this compound was tentatively assigned to a ferulate pentose ester.

To further illustrate the potency of both citric acid and sodium carbonate to release WB bound polyphenols, quantitative analysis was also performed, considering FA and FA-d. From the data given in Table 5, it could be supported that water and 60% ethanol were very poor means of recovering either FA or FA-d.

On the other hand, the treatment carried out with sodium carbonate resulted in the release of FA, which was comparable, though lower, to that achieved with alkaline hydrolysis. This finding pointed emphatically to the ability of sodium carbonate to catalyze the liberation of bound FA, and to the best of the authors’ knowledge, this is the first demonstration of such an effect accomplished under mild alkaline conditions. Likewise, the citric acid treatment was also shown to be highly effective in releasing bound FA, but in the form of a pentose ester. This result was also in line with a recent investigation, which demonstrated that FA release under alkaline conditions was overwhelmingly favored compared to acidic conditions [29].

It is known that, in WB tissues, FA is linked to cell wall arabinoxylans via ester bonds [37,38] (Figure 4). Ester bonds may be cleaved by alkaline hydrolysis, which results in increased FA release. FA may also act as a cross-linking phenolic between polysaccharides and lignins. In this case, too, FA is primarily bound to polysaccharides with alkali-labile ester bonds [39]. Ether-linked FA may be released only upon acid catalysis, a process that usually requires temperatures well above the 90 °C temperature used in this study. In the acid-catalyzed treatment, fractions of hemicellulose may be solubilized, yet the ester-linked ferulic acid is not acted upon. On the other hand, ester bonds may be cleaved even under mild alkaline conditions, if the appropriate temperature is provided [40]. Thus, the adjustment of temperature and/or time at optimum values would be critical to maximizing FA recovery, considering that in thermal processes, temperature and time are interdependent variables.

### 2.6. Antioxidant Characteristics

**Figure 5:**
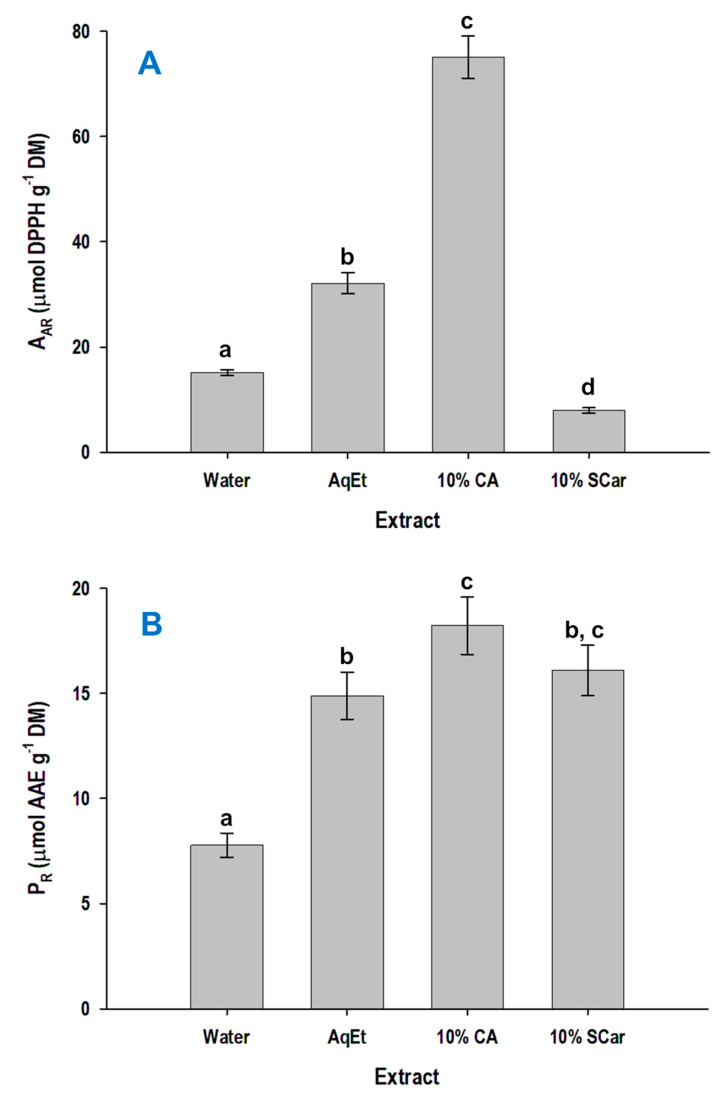
Antiradical activity (A_AR_) (**A**) and ferric-reducing power (P_R_) (**B**) of the extracts produced with hydrothermal treatment of WB, for 24 h, at 90 °C, using 10% citric acid (CA) and 10% sodium carbonate (SCar). The values of the control extracts produced with water and 60% aqueous ethanol (AqEt) are also given. Values denoted with different letters (a, b, c, and d) are statistically different (*p* < 0.05).

The extracts obtained with 10% citric acid and 10% sodium carbonate, along with the control extracts produced with deionized water and 60% ethanol were tested for their antiradical activity (A_AR_) and the ferric-reducing power (P_R_). The extract produced with 10% citric acid displayed significantly higher A_AR_ (*p* < 0.05), but the extract obtained with 10% sodium carbonate had the weakest A_AR_ (Figure 5A). On the other hand, the pattern concerning the expression of P_R_ was completely different (Figure 5B).

In this case, the extracts prepared with 10% sodium carbonate and 10% citric acid had comparable performance, although the extract produced with 10% citric acid was once again the most effective. By contrast, the extract obtained with water had very low P_R_ (*p* < 0.05).

It is irrefutable that the expression of antioxidant activity of a polyphenol mixture is rather unpredictable and depends both on the nature of the polyphenols, as well as on their relative amounts. This is because combinations of individual polyphenols may bring about synergistic and/or antagonistic effects amongst them [41,42]. Thus, it could be argued that the overall antiradical activity or ferric-reducing power consists of the integration of such phenomena. However, the antiradical activity exhibited by WB extracts has been linked, to some extent, to the polyphenol concentration [43], but earlier examinations suggested that hydrolysis under alkaline conditions, which liberates a high amount of bound phenolic acids, may show higher antioxidant activity [16]. On the other hand, in this study, it was shown that the extracts prepared with 10% citric acid, which were enriched in the ferulate pentose ester, were the most active with regard to both A_AR_ and P_R_. Thus, it could be supported that this compound might be a more powerful antioxidant compared to FA. Indeed, it has been long before demonstrated that ferulate esters may be more potent antioxidants than FA itself [44]. Such a phenomenon was also shown for ferulate glucose esters [45] and also ferulate arabinose [46]. On such a basis, it could be likely that the enhanced antioxidant effects seen for the extract obtained with 10% citric acid were attributed to the ferulate pentose ester. Yet, such an assumption remains to be elucidated.

## 3. Materials and Methods

### 3.1. Chemicals

Sodium carbonate anhydrous, L-lactic acid (80%), and sodium acetate anhydrous were from Penta (Prague, Czech Republic). 2,2-Diphenyl-1-picrylhydrazyl (DPPH) was purchased from Alfa Aesar (Karlsruhe, Germany). Acetic acid and 2,4,6-tris(2-pyridyl)-s-triazine (TPTZ) was from Fluka (Steinheim, Germany). L-Ascorbic acid was obtained from Carlo Erba (Milano, Italy). Sodium citrate tribasic dihydrate (>99%), iron chloride hexahydrate (FeCl_3_), and citric acid anhydrous were from Merck (Darmstadt, Germany). Absolute ethanol and Folin–Ciocalteu regent were from Panreac (Barcelona, Spain). Ferulic acid, vanillic acid, protocatechuic acid and p-coumaric acid were from Sigma-Aldrich (Steinheim, Germany). All solvents used for chromatography were of appropriate grade.

### 3.2. Wheat Bran

Bran originating from hard wheat (*Triticum aestivum*) processing was kindly donated by Katsaris Mills (Karditsa, Central Greece). The bran was freshly produced (24 h) and stored in a dry, dark, and well-aerated chamber, at temperatures that did not exceed 26 °C. Upon receipt, WB was pulverized in a table coffee mill and sieved to collect powder with average particle diameter <300 μm. This material was placed in air-tight containers and stored at 4 °C for no longer than 4 days.

### 3.3. Reference Alkaline Hydrolysis

The amount of polyphenols liberated upon alkaline treatment of a plant tissue is generally regarded to be the total extractable content. Thus, an alkaline hydrolysis methodology was employed to serve as a reference procedure [18]. The protocol implemented was based on a previous one [17], with modifications dictated by recent studies, pertaining to methanol incorporation into the reaction mixture and the adjustment of sodium hydroxide concentration and temperature [29]. The exact mass of 1.0 g of WB was mixed with 10 mL 60% methanol containing 2 M NaOH, and the mixture was stirred for 4 h at 40 °C. Then, 3 mL HCl (6 M) and 3 mL formic acid (1 M in methanol) were added, mixed well, and the mixture was centrifuged at 5000× *g*. The clear supernatant was filtered 0.45 μm PVDF syringe filter and used for HPLC determinations.

### 3.4. Hydrothermal Treatments

Based on data from a previous study, all treatments were performed at 90 °C [21]. This constant temperature was provided by an oil bath placed on a thermostated hotplate (Witeg, Wertheim, Germany). The extraction vial (25 mL Duran™ bottle with screw-cap closure) containing 10 mL of each aqueous system tested was placed in the oil bath for 5 min to acquire 90 °C. Then, 1 g of the pulverized WB was introduced, and the treatment was carried out for 3, 6, and 24 h, under constant stirring at 400 rpm, provided by the hotplate. The aqueous systems used for the hydrothermal treatments were mild acidic or alkaline solutions of natural organic acids or organic and inorganic salts of various pH values, as presented analytically in Table 1. After each treatment, the extracts were centrifuged for 10 min at 10,000× *g* and stored at −40 °C.

### 3.5. Process Severity Assessment

Hydrothermal severity process may be evaluated by taking into consideration the temperature and resident time, and it can be used as a criterion in assessing different treatment conditions, as follows [47,48]:

(3) $$R_{o}=t\;\times \;e^{\left(\frac{T-100}{14.75}\right)}$$

SF = *logR*_o_ (4)

The term SF corresponds to severity factor, and *R*_o_ and 100 represent the severity and the reference temperature (°C), respectively. The value 14.75 is an empirical parameter, correlated with treatment temperature and activation energy. The combined severity factor (CSF) is regarded as an extended form of SF and takes into account the pH of the hydrothermal medium (solvent) that may also be implicated in biomatrix (WB) disintegration [35]:

(5) $${R_{o}}^{\prime }=10^{-\mathrm{pH}}\;\times \;t\;\times \;e^{\left(\frac{T-100}{14.75}\right)}$$

CSF = *logR*_o_′ − pH (6)

Furthermore, the determination of the alternative CSF, termed as CSF’, is another approach on this issue which has been used in previous examinations and may provide a fairer comparison of the severities of different treatments, within large pH ranges [35]:

CSF’ = *logR*_o_ + |pH − 7| (7)

### 3.6. Determination of Total Polyphenols and Antioxidant Activity

A protocol previously described [49] was used for total polyphenol determination. The methodology was based on the Folin–Ciocalteu reagent, and ferulic acid was used as the calibrating standard (50–700 mg L^−1^, R^2^ = 0.9992). Results were expressed as mg ferulic acid equivalents (FAE) per g dry mass (DM). The antioxidant activity was estimated by determining both the antiradical activity (A_AR_) and the ferric-reducing power (P_R_), using the methodologies reported elsewhere [21]. Briefly, for the A_AR_ determination, the stable radical probe DPPH was used, and results were given as μmol DPPH per g DM. Similarly, for the P_R_ determination, the complexing agent TPTZ was used as a chromophore complexing agent for Fe^2+^, and results were expressed as μmol ascorbic acid equivalents (AAE) per g DM.

### 3.7. Liquid Chromatography–Diode Array–Mass Spectrometry (LC–DAD–MS)

The device used was a Finnigan MAT Spectra System P4000 pump (San Jose, CA, USA), in line with a UV6000LP diode array detector and a Finnigan AQA mass spectrometer. Chromatography was run on a Fortis RP-18 column, 150 mm × 2.1 mm, 3 μm, at 40 °C using a 10 μL injection. Acquisition of mass spectra was accomplished with electrospray ionization (ESI) in positive ion mode by deploying mass spectrometer settings and elution conditions described elsewhere [50]. Quantification was performed with an external standard, using a calibration curve of ferulic acid (R^2^ = 0.9977), and standard solutions with concentrations ranging from 0 to 50 μg mL^−1^ (R^2^ = 0.9980). Standard solutions were prepared in HPLC grade methanol shortly prior to analyses.

### 3.8. Statistical Processing

Each extraction process was performed at least twice, and every determination was carried out in triplicate. The results reported represent mean values ± standard deviation. Linear regressions were accomplished with SigmaPlot™ v.15 (Systat Software Inc., San Jose, CA, USA). Data normality was investigated using the Shapiro–Wilk test. Considering that the data were not normally distributed, statistically significant differences were revealed with the Kruskal–Wallis test, using IBM SPSS Statistics™ 29 (SPSS Inc., Chicago, IL, USA).

## 4. Conclusions

In this study, a series of organic acids, organic acid salts, and sodium carbonate, were tested for their ability to provide mild acidic and alkaline conditions and catalyze the hydrolysis of WB-bound polyphenols during hydrothermal treatment. The outcome of the investigation clearly demonstrated that both citric acid and sodium carbonate may be used as effective acid and alkali catalysts, respectively, to achieve high polyphenol recovery yields. The assessment of the processes employed using the severity factor also showed that the treatments with either citric acid or sodium carbonate could be performed under conditions that provided comparable severity. However, the thorough examination of the extracts produced with either catalyst gave sound evidence of a differentiated hydrolysis mechanism, which resulted in extracts with diversified composition. The extract obtained from the citric-acid-catalyzed treatment was found to be enriched in a ferulic acid derivative, tentatively identified as a ferulate pentose ester, and exhibited increased antioxidant activity compared to the sodium carbonate extract, which was dominated by ferulic acid. The information emerging from this study suggests emphatically that both citric acid and sodium carbonate may be used as highly efficient benign catalysts to obtain exceptionally increased polyphenol recoveries, through the hydrothermal treatment of WB. Such a property might enable the establishment of sustainable techniques of extraction, using environmentally friendly and food-compatible chemicals, instead of the non-green sulfuric acid and sodium hydroxide, to achieve equivalent yields. Such processes, in turn, could be integrated in wider strategies of plant biomass biorefining.

## Figures and Tables

**Table 1:** Natural organic acid, organic acid salt, and sodium carbonate solutions used in this study. Information on the concentration (both % and molar), as well as the pH of each solution employed, are also given.

Extraction Medium	*C* (% *w*/*v*)	*C_mol_* (mol L^−1^)	pH
Acetic acid	0.5	0.083	2.46
	1.0	0.167	2.40
	2.5	0.416	2.37
	5.0	0.833	2.33
Lactic acid	0.5	0.056	2.70
	1.0	0.111	2.67
	2.5	0.278	2.40
	5.0	0.555	2.10
Citric acid	0.5	0.026	2.73
	1.0	0.052	2.52
	2.5	0.130	2.36
	5.0	0.260	2.19
Sodium carbonate	0.5	0.047	11.25
	1.0	0.094	11.34
	2.5	0.236	11.39
	5.0	0.472	11.49
Sodium acetate	0.5	0.061	7.35
	1.0	0.122	7.64
	2.5	0.305	7.88
	5.0	0.610	8.90
Trisodium citrate	0.5	0.019	7.98
	1.0	0.039	8.12
	2.5	0.097	8.58
	5.0	0.194	8.62

**Table 2:** Yield in total polyphenols (Y_TP_) achieved after hydrothermal treatment of WB with each of the acid solutions tested. Measurements were carried out after 3, 6, and 24 h of treatment, and results are given as means ± standard deviation (n = 3).

Solvent (% *w*/*v*)	Y_TP_ (mg FAE g^−1^ DM)					
	3 h	sd	6 h	sd	24 h	sd
Water	3.33 ^a^	0.09	3.61 ^a^	0.05	3.25 ^a^	0.03
Acetic acid						
0.5	2.78 ^b^	0.04	2.64 ^b^	0.08	2.94 ^b^	0.04
1.0	2.67 ^b^	0.07	2.85 ^b^	0.07	3.26 ^a^	0.05
2.5	3.31 ^a^	0.06	3.36 ^c^	0.05	3.57 ^c^	0.06
5.0	3.78 ^c^	0.10	3.93 ^d^	0.10	4.13 ^d^	0.03
Lactic acid						
0.5	2.73 ^b^	0.04	2.91 ^b^	0.08	3.58 ^c^	0.07
1.0	3.00 ^d^	0.06	3.43 ^c^	0.05	4.19 ^d^	0.03
2.5	3.05 ^d^	0.05	3.98 ^d^	0.04	4.65 ^e^	0.04
5.0	4.22 ^e^	0.12	4.58 ^e^	0.11	5.82 ^f^	0.08
Citric acid						
0.5	2.81 ^b^	0.11	4.01 ^d^	0.11	5.49 ^g^	0.05
1.0	3.33 ^a^	0.06	3.52 ^a^	0.07	5.49 ^g^	0.10
2.5	3.76 ^c^	0.05	4.48 ^e^	0.09	9.27 ^h^	0.12
5.0	5.09 ^f^	0.11	6.07 ^f^	0.11	18.77 ^i^	0.14

Values reported are means (n = 3) ± standard deviation. Values within columns denoted with different letters (a, b, c, d, e, f, g, h, and i) are statistically different (*p* < 0.05).

**Table 3:** Yield in total polyphenols (Y_TP_) achieved after hydrothermal treatment of WB with each of the salt solutions tested. Measurements were carried out after 3, 6, and 24 h of treatment, and results are given along with standard deviation (sd).

Solvent (% *w*/*v*)	Y_TP_ (mg FAE g^−1^ DM)					
	3 h	sd	6 h	sd	24 h	sd
Water	3.33 ^a^	0.07	3.61 ^a^	0.01	3.25 ^a^	0.03
Sodium carbonate						
0.5	10.48 ^b^	0.05	13.01 ^b^	0.26	12.61 ^b^	0.02
1.0	14.98 ^c^	0.22	13.39 ^b^	0.20	14.41 ^c^	0.20
2.5	13.64 ^d^	0.12	17.79 ^c^	0.19	20.84 ^d^	0.40
5.0	15.72 ^e^	0.14	19.38 ^d^	0.21	22.33 ^e^	0.31
Sodium acetate						
0.5	2.44 ^f^	0.06	1.76 ^e^	0.05	2.84 ^f^	0.11
1.0	1.54 ^g^	0.06	2.42 ^f^	0.07	4.17 ^g^	0.14
2.5	2.51 ^f^	0.10	2.46 ^f^	0.01	3.90 ^a^	0.10
5.0	2.77 ^h^	0.06	3.06 ^g^	0.09	4.19 ^g^	0.03
Sodium citrate tribasic						
0.5	3.22 ^a^	0.08	3.47 ^a^	0.14	5.39 ^h^	0.02
1.0	5.86 ^i^	0.01	6.58 ^h^	0.12	3.33 ^a^	0.04
2.5	2.49 ^f^	0.04	2.63 ^i^	0.01	3.36 ^a^	0.11
5.0	1.61 ^g^	0.07	1.16 ^j^	0.09	2.69 ^f^	0.09

Values reported are means (n = 3) ± standard deviation. Values within columns denoted with different letters (a, b, c, d, e, f, g, h, i, and j) are statistically different (*p* < 0.05).

**Table 4:** The alternative severity factor (CSF’) determined for the hydrothermal treatments performed with citric acid and sodium carbonate solutions (2.5–10%), along with the corresponding yields in total polyphenols (Y_TP_). All treatments were performed at 90 °C.

*C* (% *w*/*v*)	*t* (min)	CSF’		Y_TP_ (mg FAE g^−1^ DM)			
		CA	SCar	CA	sd	SCar	sd
2.5	180	6.60	6.30	3.76 ^a^	0.05	13.64 ^a^	0.12
	300	6.90	6.60	4.48 ^b^	0.09	17.79 ^b^	0.19
	1440	7.50	7.20	9.27 ^c^	0.12	20.84 ^c^	0.40
5.0	180	6.77	6.31	5.09 ^d^	0.11	15.72 ^d^	0.14
	300	7.07	6.61	6.07 ^e^	0.11	19.38 ^c^	0.21
	1440	7.67	7.21	18.77 ^f^	0.14	22.33 ^e^	0.31
10.0	180	7.47	6.64	6.16 ^e^	0.09	17.24 ^b^	0.11
	300	7.77	6.94	8.93 ^c^	0.17	19.65 ^c^	0.14
	1440	8.37	7.54	23.76 ^g^	0.32	23.60 ^e^	0.19

Values within columns denoted with different letters (a, b, c, d, e, f, and g) are statistically different (*p* < 0.05).

**Table 5:** Polyphenolic composition of extracts obtained with hydrothermal treatment of WB, using either citric acid or sodium carbonate as catalysts.

Extraction Medium	Extraction Yield (μg g^−1^ DM) *		
	Ferulic Acid	Ferulate Derivative	Total
Alkaline hydrolysis	2158.61 ± 112.02 ^a^	210.44 ± 5.43 ^a^	2369.05 ^a^
Water	37.22 ± 2.65 ^b^	18.83 ± 0.50 ^b^	56.06 ^b^
60% (*v*/*v*) Ehanol	32.62 ± 2.52 ^b^	19.16 ± 1.56 ^b^	55.79 ^b^
10% (*w*/*v*) Citric acid	344.52 ± 3.55 ^c^	1930.70 ± 58.46 ^c^	2275.22 ^c^
10% (*w*/*v*) Sodium carbonate	1822.97 ± 16.66 ^d^	232.04 ± 2.78 ^d^	2055.01 ^d^

* Values reported are means (n = 3) ± standard deviation. Values within columns denoted with different letters (a, b, c, and d) are statistically different (*p* < 0.05).

## Data Availability

The data presented in this study are available on request from the corresponding author. The data are not publicly available due to confidentiality required to continue the study.
